# Discovery of Avian Paramyxoviruses APMV-1 and APMV-6 in Shorebirds and Waterfowl in Southern Ukraine

**DOI:** 10.3390/v15030699

**Published:** 2023-03-08

**Authors:** Amy C. Klink, Oleksandr Rula, Mykola Sushko, Maksym Bezymennyi, Oleksandr Mezinov, Oleksandr Gaidash, Xiao Bai, Anton Stegniy, Maryna Sapachova, Roman Datsenko, Sergiy Skorokhod, Vitalii Nedosekov, Nichola J. Hill, Levan Ninua, Ganna Kovalenko, Anne Lise Ducluzeau, Andriy Mezhenskyi, Jeremy Buttler, Devin M. Drown, Douglas Causey, Borys Stegniy, Anton Gerilovych, Eric Bortz, Denys Muzyka

**Affiliations:** 1 Department of Biological Sciences, University of Alaska Anchorage, Anchorage, AK 99508, USA; 2 National Scientific Center Institute of Experimental and Clinical Veterinary Medicine, 61023 Kharkiv, Ukraine; 3 State Scientific and Research Institute of Laboratory Diagnostics and Veterinary and Sanitary Expertise, 03151 Kyiv, Ukraine; 4 Institute for Veterinary Medicine, National Academy of Agrarian Sciences, 03151 Kyiv, Ukraine; 5 The F.E. Falz-Fein Biosphere Reserve “Askania Nova”, Askania-Nova, 75230 Kakhovka Raion, Ukraine; 6 Institute of Natural Sciences, Department of Zoology, H.S. Skovoroda Kharkiv National Pedagogical University, 61022 Kharkiv, Ukraine; 7 Danube Biosphere Reserve, National Academy of Sciences of Ukraine, 68355 Vilkove, Ukraine; 8 Department of Epizootology, The National University of Life and Environmental Science of Ukraine, 03041 Kyiv, Ukraine; 9 Department of Biology, University of Massachusetts, Boston, MA 02125, USA; 10 Institute of Ecology, Ilia State University, Tbilisi 0162, Georgia; 11 Division of Virology, Department of Pathology, University of Cambridge, Cambridge CB2 1QP, UK; 12 Department of Biology and Wildlife, University of Alaska Fairbanks, Fairbanks, AK 99775, USA

**Keywords:** viral ecology, surveillance of avian paramyxoviruses, APMV, wild birds, next-generation sequencing, minion, Azov-Black Sea region in Ukraine

## Abstract

Emerging RNA virus infections are a growing concern among domestic poultry industries due to the severe impact they can have on flock health and economic livelihoods. Avian paramyxoviruses (APMV; avulaviruses, AaV) are pathogenic, negative-sense RNA viruses that cause serious infections in the respiratory and central nervous systems. APMV was detected in multiple avian species during the 2017 wild bird migration season in Ukraine and studied using PCR, virus isolation, and sequencing. Of 4090 wild bird samples collected, mostly from southern Ukraine, eleven isolates were grown in ovo and identified for APMV serotype by hemagglutinin inhibition test as: APMV-1, APMV-4, APMV-6, and APMV-7. To build One Health’s capacity to characterize APMV virulence and analyze the potential risks of spillover to immunologically naïve populations, we sequenced virus genomes in veterinary research labs in Ukraine using a nanopore (MinION) platform. RNA was extracted and amplified using a multiplex tiling primer approach to specifically capture full-length APMV-1 (*n* = 5) and APMV-6 (*n* = 2) genomes at high read depth. All APMV-1 and APMV-6 fusion (F) proteins possessed a monobasic cleavage site, suggesting these APMVs were likely low virulence, annually circulating strains. Utilization of this low-cost method will identify gaps in viral evolution and circulation in this understudied but important critical region for Eurasia.

## 1. Introduction

Avian paramyxoviruses (family Paramyxoviridae; also known as avian avulavirus) are a highly diverse group of zoonotic, negative-sense, single-stranded RNA viruses detected in a variety of domestic and wildlife species. Avian paramyxoviruses (APMV) range in genomes sizes from 13–17 kilobases (kb) long, comprising 22 unique subtypes or serotypes (1–22) classified into three distinct genera according to the International Committee on Taxonomy of Viruses: metaavulavirus (serotypes – 2, 5, 6, 7, 8, 10, 11, 14, 15, 20, 21, 22), orthoavulavirus (serotypes – 1, 9, 12, 13, 16, 17, 18, 19), and paraavulavirus (serotypes – 3, 4). Among these species of viruses is avian orthoavulavirus serotype 1 (APMV-1) that commonly induces Newcastle disease [1]. In previously published research, APMV-1 was commonly referred to as Newcastle disease virus (NDV), particularly when found in domestic poultry, while APMV-1 was commonly characterized in wild birds [1]. The pathogenicity of APMV-1 or NDV ranges from velogenic (high), mesogenic (mild), to lentogenic (low) depending on the strain, serotype, infected host, and specific molecular characteristics of the proteolytic cleavage site located in the fusion protein [2]. Severe avulaviruses infections (mainly for APMV-1 or NDV) cause Newcastle disease (ND) in poultry, with symptoms of depression, conjunctiva, mucosal excretions from the crop, green diarrhea, hemorrhages, necrosis, and misshapen egg production [3]. 

Newcastle disease is an emergent disease and very important for the poultry industry, which typically induces neurotropic symptoms associated with velogenic ND, including lethargy, muscle tremors, paralysis, and ultimately death [3]. APMV-1 or NDV has been shown to elicit severe symptoms and is of the highest concern because of the deleterious effects it can have on the poultry industry [3,4]. APMV is found worldwide among a variety of species; however, each serotype will elicit a suite of symptoms depending on the species infected. The term “serotype” is in common usage for describing APMV subtypes (genetic groups), but recently sequencing rather than serological analysis has become the main technique for identifying APMV strains. Serotype or subtype assignment is largely based on phylogenetics [1,5,6,7]. Indeed the genetic classification system for APMV, which previously relied on serological identification and amino acid sequence of large (L) polymerase protein, was updated to reflect the ability to better capture genetic diversity through phylogenetic analysis of complete genomes [7,8].

APMV of different subtypes is commonly isolated from poultry and other domesticated birds, although wildlife hosts have been described [5,6,7,9,10,11,12]. APMV-1 is the most diverse of the subtypes and is classified into two distinct classes (class I or class II) and further characterized into either 1 (class I) or 15 genotypes (class II) [9]. Class I viruses are solely isolated from wild birds (e.g., non-farmed birds), while class II viruses encompass poultry, domesticated, and wild captive populations [9,13]. Little is known about the effects of other APMV subtypes in wildlife vectors due to the limited amount of data collected.

Aside from APMV-1, other subtypes of avulavirus are less prevalent in domestic birds and poultry and are instead typically isolated from wild birds. Among these, avian meta-avualavirus 6 (APMV-6) has been described to cause mild respiratory infection as well as reproductive implications in poultry and turkeys [3,14,15]. The first description of APMV-6 was isolated from a domestic duck in Hong Kong in 1977 [14]. APMV-6 can be characterized into two distinct subgroups based on both the antigenic serotype and genomic make-up; however, only 38 complete genomes have been sequenced prior to this study, so the actual diversity of this serotype in nature is unknown [14,16]. APMV-6 is genetically different from other APMV subtypes because it encodes a small hydrophobic (SH) protein. The SH protein has been identified in other paramyxoviruses, with a unique function for the associated virion [14]. The function of the SH protein in APMV-6 is still unknown, although APMV-6 was hypothesized to be the oldest ancestor of all avian paramyxoviruses because of the similarity to the SH protein as seen with other viruses in the *Paramyxoviridae* family. 

The diversity of avian paramyxoviruses is widespread among hosts, but there is a large gap in knowledge and understanding of the movement and evolution of these viruses within wild populations [8]. A large portion of the data collected is dependent on existing bird sampling efforts for other viruses (e.g., avian influenza viruses, or AIV) with a consequent bias towards geographical regions.

Ukraine is a hot spot for the detection of avian diseases in wild bird populations because the geographical region spans three major flyways: East Africa and West Asia, Central Asia, and the Black and Mediterranean Seas. Similar to other RNA viruses (e.g., AIV), the introduction of APMV to naïve poultry populations is thought to be transmitted from wild migratory birds [6,7,9,11,16]. Horizontal transmission occurs through oropharyngeal and fecal secretions, thus resulting in a facile route of infection. Birds are highly gregarious and inhabit similar physical locations, so the potential for transmitting viral pathogens and commensal microbes is high [17]. APMV and especially APMV-1, as the causative agent of ND, are important pathogens to control in the Ukrainian poultry industry to maintain a healthy and thriving poultry industry. Importantly, APMV-1 outbreaks in poultry occur worldwide, so understanding APMV-1 subtypes and pathotypes have the potential to contribute to a deeper understanding of APMV distribution and evolution worldwide.

Broad wild bird surveillance for AIV and APMV was conducted from 2006 to 2016 in Ukraine, in regions observed to be on intercontinental flyways for wild birds in Eurasia (North–South and East–West flyways). A total of 21,511 samples were collected from 105 species of wild birds representing 27 families and 11 orders. Eighty-two low pathogenic avian influenza (LPAI) viruses were isolated from wild birds with a total of 23 antigenic hemagglutinin (HA) and neuraminidase (NA) combinations [18]. Fifteen of the sixteen known avian influenza HA subtypes were isolated. Five H5N8 highly pathogenic avian influenza (HPAI) viruses and two H5N2 LPAI viruses were isolated from live wild birds and environmental samples (fresh bird feces) from samples collected for surveillance to understand the risks of AIV and APMV outbreaks in poultry [19,20].

In Ukraine, over 154 AIV have been isolated, including 14 AIV hemagglutinin subtypes detected among 66 wild bird species [18,19,20]. In previous Ukrainian bird surveillance efforts, APMV-1, APMV-4, APMV-6, and APMV-7 were detected by serological analyses of APMV isolated from wild bird samples; however, the genetic sequences and severity of the associated diseases have been poorly understood. From 2006-2011, twenty APMV viruses were isolated and serologically identified as APMV-1 (*n*= 9), APMV-4 (*n* = 4), APMV-6 (*n* = 3), and APMV-7 (*n* = 4) in Ukraine from wild birds from 2006–2011 [12]. Recently in Ukraine, the diagnosis of APMV and NDV has relied on PCR, virology, and serological analysis; however, it can be difficult to distinguish between velogenic and lentogenic strains of APMV without the whole genome sequence [3]. Sequencing APMV genomes can alleviate problems with the ambiguity of serological assays and identify genetic characteristics important for pathotyping [9]. To build a genomic sequencing capacity for avian pathogens, we developed protocols for and deployed the Oxford Nanopore Technologies (ONT) MinION portable nanopore sequencing platform in sentinel veterinary health labs in Ukraine. MinION has revolutionized avian disease surveillance efforts in low-resource regions that previously relied on sending samples to high throughput sequencing labs at a high cost [21].

Ukraine relies on the agricultural industry, which has an important poultry component (235–250 million birds) divided between industrial poultry and backyard flocks. The impacts of Newcastle disease outbreaks on the agricultural industry are large and can affect the economy [22]. For this reason, surveillance, isolation, and sequencing of APMV of different subtypes are very important for the persistence of Ukraine’s and Europe’s agricultural industries. Thus, we isolated and sequenced novel APMV-1 and APMV-6 from wild birds in Ukraine and analyzed the phylogenetic relationships of these pathogens with known isolates of APMV from Eurasia.

## 2. Materials and Methods

### 2.1. Sample Collection

**Figure 1:**
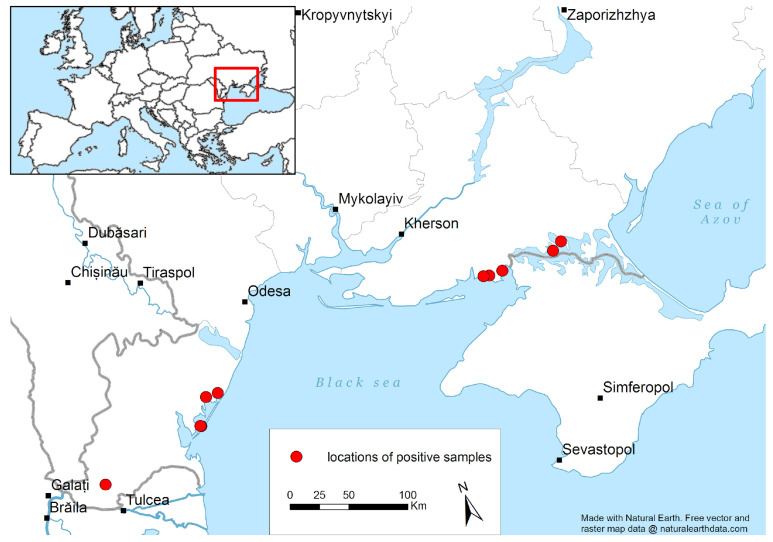
Map of locations of samples that were positive for avian paramyxoviruses in southern Ukraine. Environmental (feces) samples from wild birds were collected in southern Ukraine (2016–2017) in Kherson, Odesa, Mykolaiv, and Zaporizhzhya *Oblasts*. Locations where samples tested positive for avian paramyxoviruses by diagnostic RT-PCR are indicated (•). Positive wild bird samples were found in proximity to brackish or salt water *liman* and Sivash (bays) in Kherson *Oblast* near Crimea and in proximity to Sasyk L(lagoons) and the Danube River Delta region in southern Odesa *Oblast*.

Wild bird surveillance was conducted from December 2016 to December 2017 in the northern and southern regions of Ukraine (Figure 1). The research was conducted under an approved IACUC protocol (NSC IECVM UP-4 IACUC and ethical review, protocols approved: 18 January 2017; #1–19, 20 February 2019; #2–19, 22 February 2019). In cooperation with ornithologists who helped identify bird species, 4790 fecal (environmental) samples were collected from 40 species of wild birds (Table S1). Sampling from the wild birds was conducted according to the standard operating procedures (SOP) describing biosafety measures for collectors, sample collection and preservation, cryopreservation (liquid nitrogen), and overland transport. Sample collection was dependent on flock size; at least 25 samples per 500 birds in the flock and at least 50 samples per 1000 birds in the flock. Feces were collected only if the origin and type of bird were established. Samples of feces were taken in a checkerboard pattern at a distance of at least 1.5–2 m from each other to avoid selecting feces from the same bird. A total of 4315 fecal samples were collected from the Azov-Black Sea region of Southern Ukraine (Kherson, Odesa, Mykolaiv, Zaporizhzhya Oblasts), one of the most important regions in Eastern Europe regarding wild birds of different ecological groups. A total of 475 fecal samples were obtained from wild birds in northern Ukraine (Chernihiv Oblast).

Samples were collected in cryotubes containing 1.0 mL of viral transport media (BHI, Brain Heart Infusion broth, Sigma-Aldrich, #53286-100G) with antibiotics (penicillin 10,000 U/mL, streptomycin 10 mg/mL, gentamicin 250 µg/mL, and nystatin 5000 U/mL). Commercially available powdered concentrates were prepared and sterilized immediately prior to use [23]. Fecal samples were stored at −196° C in liquid nitrogen, where they were kept until processing.

### 2.2. Virus Isolation

Virus isolation from the fecal samples was conducted in accordance with typical OIE procedures [24,25]. Fecal samples were inoculated in the allantoic cavity of 9–10 day old embryonated chicken eggs and passaged three times in ovo. The presence of hemagglutinating (HA) viruses in the allantoic fluid was determined by a hemagglutinin inhibition (HI) test with a 1% suspension of chicken red blood cells [24,25]. Samples from PCR-positive pools were individually retested to identify positive sample(s).

### 2.3. Virus Identification

The HA virus subtype was determined by HI tests (previously described, [24,25,26]). For these studies, the following antisera were used: H1N1, H2N3, H3N8, H4N6, H5N1, H6N8, H7N1, H8N4, H9N2, H10N7, H10N9, H11N6, H12N5, H13N6, H14N6, H15N9, H16N3, APMV-1, APMV-2, APMV-3, APMV-4, APMV-6, APMV-7, APMV-8, and APMV-9 produced by the Veterinary Laboratories Agency (Animal and Plant Health Agency, Weybridge, UK). The antisera to H1N1, H2N3, H3N8, H4N8, H5N3, H6N2, H7N3, H8N4, H9N7, H10N1, H11N9, H12N5, H13N6, H14N5, H15N9, H16N3, APMV-1, APMV -2, APMV-3, APMV-4, APMV-6, APMV-7, APMV-8, and APMV-9 were produced by the Instituto Zooprofilattio Sperimentale delle Venezie, Padova, Italy. Positive sera produced at NSC IECVM (Kharkiv, Ukraine) against HPAI virus A/chicken/Syvash/02/2005 (H5N1) and LPAI virus A/teal/Djankoy/4-17-11/2010 (H5N2) were also used.

### 2.4. RNA Extraction and Diagnostic RT-PCR for APMV

Pooled samples were analyzed by real-time reverse transcription PCR (RT-PCR). RNA extraction was performed using QIAamp Viral RNA Mini Kit (QIAGEN). Extracted RNA was stored at −70 °C and used for amplification and detection of APMV-1 in one-step RT-PCR using AgPath-ID™ one-step RT-PCR Reagents (Applied Biosystems). The RT-PCR analysis was qualitative by analysis of cycle threshold (C_t_) values, with C_t_ value < 45 considered positive. Detection of APMV-1 by qRT-PCR was conducted using AgPath-ID™ One-Step RT-PCR reagents and specific primers according to Czegledi et al. (2006) [27]. The following primers and probe were employed for qRT-PCR [28]: forward primer M+4100: 5′-AGTGATGTGCTCGGACCTTC-3′; reverse primer M-4220: 5′-CCTGAGGAGAGGCATTTGCTA-3′; probe M+4169: 5′-[FAM]TTCTCTAGCAGTGGGACAGCCTGC[TAMRA]-3′ All samples from APMV-1 positive pools were tested by the same method.

### 2.5. Dataset Development for Genome Assembly

Datasets were created to assemble sequencing reads as well as to reconstruct phylogenetic relationships. Sequences were downloaded from the Virus Pathogen Resource (ViPR, at https://www.viprbrc.org/, accessed on 5 May 2022) or NCBI GenBank (https://www.ncbi.nlm.nih.gov/, accessed on 5 May 2022) and were specified to include full-length viral genomes. Vaccine strains and duplicated sequences were removed from the dataset. Metadata was collected for each sequence from GenBank or ViPR, including the following fields: host (common name), sample location, and sample collection date. Representative sequences from each serotype and subtype were compiled for preliminary analysis, and genome assembly was conducted as described above.

### 2.6. Development of Tiling Primers for Full Genome Amplification

Due to the variable viral load in each sample, the RNA was amplified to maximize sequencing efficiency. Viral RNA was amplified in either 1000 nucleotides (nt) or 1500 nt regions generated by spanning the viral genome with a 50 or 200 nt overlap using the Primal Scheme program (http://primal.zibraproject.org/, accessed on 5 May 2022) [29]. The sequences presented here utilized both types of schemes (1000 nt region with 50 nt overlap; 1500 nt region with 200 nt overlap); however, schemes with a 1500 nt region or 200 nt overlap were more efficient and cost-effective for amplification (Table S5; .bed files available upon request, which were used to mask primer sequences during genome assembly steps). Primers (reverse and forward) were pooled into four pools according to primal scheme output.

### 2.7. Amplicon Synthesis, cDNA Library Preparation, and Bioinformatics

APMV genome sequencing allowed the simultaneous identification of serotype (subtype) and evaluation of pathotype of the virus. Previous sequencing of virus genomes has relied on resource-intensive Illumina or Sanger sequencing. With the development of the Oxford Nanopore Technologies’ (ONT) third generation sequencer, MinION, sequencing has become portable, inexpensive, and able to produce highly accurate, real-time data within hours [30].

The RNA was amplified using the Superscript III One Step protocol (Invitrogen) following the manufacturer’s instructions with the tiling primers described above and the following thermocycling conditions: 2 min at 55 °C, 60 min at 42 °C, 2 min at 94 °C, 39 cycles of 30 s at 94 °C, 35 s at 55 °C, and 2 min at 68 °C. The final elongation was set for 5 min at 68 °C. SPRI bead clean-up, using Agencourt AMPure XP beads (1:1 sample to beads), removed impurities from the PCR amplicon preparations for subsequent sequencing steps. All samples were barcoded using an ONT Native Barcoding kit (EXP-NBD 104) in equimolar concentrations according to the manufacturer’s protocol. The cDNA library was prepared using an ONT genomic sequencing ligation kit (SQK-LSK 109) and sequenced for 48 h on a FLO-MIN 106 flow cell (R9.4.1) using a MinION Mk1B device.

All reads were basecalled using Guppy v3.4.4 (ONT) default parameters. Reads were demultiplexed, and barcodes were trimmed using Guppy v3.4.4 (ONT; guppy_barcode) default parameters. Reads were filtered for quality with a q score ≥10 (min_mean_q 90) and a length ≥200 bp (min_length 200) using Filtlong v.3.0 (https://github.com/rrwick/Filtlong, accessed on 5 May 2022). Samples with more than 800,000 reads were downsampled to facilitate the computational analysis by increasing the min_length value based on the N50 score. AMPV subtype (serotype) assignment was confirmed by mapping reads to a reference database containing representative sequences from each APMV subtype (Table S6) using Minimap2 v2.17 [31]. We also validated this assignment by sequence alignment of APMV L protein using BLAST. Following initial read-based subtyping, reads were subsequently mapped to a subtype-specific database (e.g., all published APMV-1 or APMV-6 genomes, respectively as described above in Section 2.5) using Minimap2 (default parameters) to identify the closest reference genome match [31]. Reads were re-mapped to the APMV reference genome with the most hits using Minimap2 and further analyzed with bedtools v2.29.2 to identify the coverage of reads across the genome of each segment [31,32]. Medaka (model r941_min_high) v0.11.5 was used to generate a consensus sequence, and filtered reads were used as the input. The reference sequence specified earlier was used as the reference scaffold.

Genomes were assembled by aligning the consensus sequence to the NCBI reference sequence GenBank file (containing protein features) using Muscle pairwise aligner v.3.8.425 with default parameters through Geneious v.11.0.3 program [33]. Genome assemblies indicated similar sizes than typical for APMV-1 and APMV-6 and generally >95% genome coverage, with a small number of ambiguities in some sequences (Figure S1 and Figure S2; Table S7). Homopolymers, due to sequencing errors were identified in each sample, were manually deleted: APMV-1/Mallard/Myt Kherson/1-4/4-09/17 had 1 manual deletion, APMV-1/Mallard/AN Kherson/TM434778/2002 had 4 manual deletions, APMV-6/Environmental/ND Kherson/41-45/7-08/17 had 2 manual deletions; APMV-6/Mallard/ND Kherson/11-15/4-09/17 had 1 manual deletion; APMV-1/mallard/Dr Kherson /1-3/5-09/17 had 3 manual deletions, APMV-1/Grey goose/Myt/1-4/4-09/17 had 2 manual deletions, and APMV-1/Shelduck/Chur/1-5/2-11/17 had 4 manual deletions (Tables S8–S14). We tested the primer amplification, nanopore MinION sequencing, and bioinformatics assembly protocols using a lab-grown vaccine strain of Newcastle disease virus (NDV/APMV-1) La Sota (a kind gift of Dr. Adolfo García-Sastre, Icahn School of Medicine at Mount Sinai, NY), and captured >99% identity to the NDV reference strain (AF077761.1).

### 2.8. Phylogenetic Analysis

Phylogenetic analysis was used to analyze the evolutionary relationships within and between the APMV-1 and APMV-6 genomes. Sequences were stratified according to APMV group, host species, year, and location (lowest administrative unit) based on information contained in the strain name. Downsampling of the published APMV genomes was performed to allow for a maximum of one sequence for each combination of APMV group, host, year, and location, resulting in a final dataset of 322 sequences representing genomes from all 14 subtypes. The sequences were aligned using MAFFT v.7.450-1 with auto alignment methods and default parameters [34,35,36]. Alignment was manually edited to eliminate exogenous features altering the biological integrity of the alignment using Jalview v2.11.0 [37]. RAxML (Randomized Axelerated Maximum Likelihood) v.8.2.4 with high-performance computing (HPC) was used to reconstruct maximum likelihood trees using a generalized time reversible (GTR) substitution model with a gamma rate of heterogeneity and a 99 random seed parsimony [38]. The significance of trees was tested by bootstrap support analysis (100 iterations). The bestTree output with the highest likelihood support was visualized using FigTree v.1.4.4, specifying a midpoint tree root and a decreasing node order.

In addition, evolutionary relationships among avian avulavirus sequences were inferred using the time-scaled Bayesian approach using BEAST v1.10.4 [39]. An HKY nucleotide substitution model, constant coalescent, and uncorrelated relaxed clock models were used [40,41]. Four independent Markov Chain Monte Carlo runs were performed, each having 300,000,000 states and sampling every 30,000 states, to generate trees and posterior probabilities of nodes.

Potential recombination in APMV-1 and APMV-6 genomes were analyzed using RDP5 for linear sequences input from the MAFFT alignments, with the following custom settings: BootScan (200 bootstrap replicates, 95% cutoff), SciScan, 3Seq, LARD model to estimate base frequencies, recombination rates (LDHAT) with a minor allele cutoff for AMPV-1 (0.17) and AMPV-6 (0.25), and tree generation, using MrBayes, was performed as previously described [42]. 

## 3. Results

### 3.1. Avian Avulavirus Detection in Wild Birds in Ukraine

From December 2016 to December 2017, 4790 fresh fecal specimens were collected from the environment in proximity to wild birds, in conjunction with ornithological surveys of avian populations, ecology, and disease surveillance. Samples were immediately stored in liquid nitrogen and shipped to veterinary laboratories for screening of avian influenza and paramyxoviruses by diagnostic RT-PCR and virus isolation in ovo. To uncover the host species diversity of AIV and APMV in Ukraine, a broad selection of samples was collected from 40 avian species from seven different orders: Anseriformes, Charadriiforms, Podicipediformes, Gruiformes, Ciconiiformes, Pelecaniformes, and Falconiformes (Table S1). Samples were collected across avian migratory periods in the Azov-Black Sea region of Ukraine and in the north, and during the fall migration (August–October), wintering (November–February), spring migration (March–May), and during localized movement from June–July that typically occurs after the nesting period.

Eleven samples collected from Odesa (*n* = 5) and Kherson (*n* = 6) in southern Ukraine tested positive for APMV-1 by diagnostic RT-PCR (Figure 1). The APMV-1 infection rate (by RT-PCR testing) in wild birds was 0.23%, varying from 0.09% to 2.43% (Table S2). A majority of samples that were PCR-positive for APMV-1 were obtained from waterfowl, including the white-fronted goose (*Anser albifrons*) (*n* = 1), mallard duck (*Anas platyrhynchos*) (*n* = 4), whooper swan (*Cygnus cygnus*) (*n* = 1), common shelduck (*Tadorna tadorna*) (*n* = 2), white pelican (*Pelecanus onocrotalus, n* = 1), and shorebirds, including a Mediterranean gull (*Larus melanocephalus*; *n* = 1), and a snipe (*Gallinago gallinago*; *n* = 1) (Table S2). No samples tested positive in northern Ukraine (Chernihiv Oblast).

Among the wild bird fecal samples that tested positive by RT-PCR for AIV or APMV, 40 hemagglutinating isolates were amplified by virus isolation in ovo and serotyped by a hemagglutination inhibition (HI) assay. HI positives included avian influenza (*n* = 19) and avian paramyxovirus (*n* = 11), including those also identified by RT-PCR diagnostic assays. In 2017, the isolation rate by virus inoculation in ovo of APMV among the screened samples varied from 0.26% to 1.94%, with the highest rate of isolation in the ruddy shelduck (*Tadorna ferruginea*) (Table S3). Among the 11 avulavirus isolates, APMV-1 (*n* = 6), APMV-4 (*n* = 2), APMV-6 (*n* = 2), and APMV-7 (*n* = 1) were serotyped by HI assays (Table S4). One sample showed evidence of both APMV-1 and APMV-7 serotypes, but we were unable to obtain sequence data to confirm a mixed infection. A portion of positively hemagglutinating samples was unidentifiable in our serological assays (*n* = 10: Table S4).

### 3.2. Sequencing and Phylogenetic Analysis

**Figure 2:**
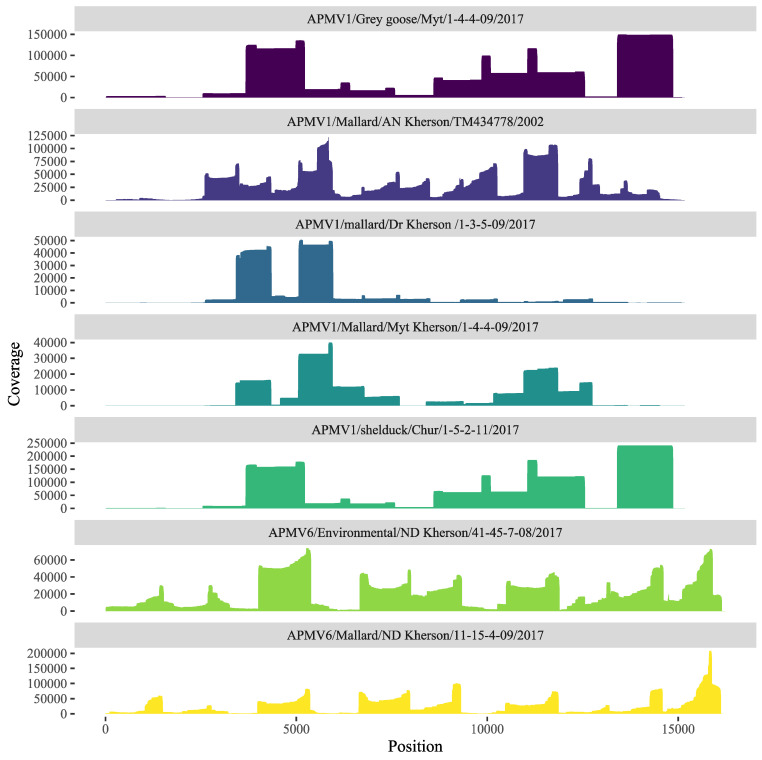
Sequencing coverage depth across full-length genomes for the seven APMV samples sequenced in this study. Viral RNA was amplified by RT-PCR with a panel of tiling primers covering the whole genome, and cDNA amplicons were sequenced using MinION. Reads were mapped to a reference, and coverage depth was calculated with the bedtools genomic arithmetic package.

The genomes of five new APMV-1 and two new APMV-6 isolates were constructed by reference-based assembly with a range in sizes from 14,098 to 16,235 nt, consistent with the average sizes of both APMV-1 and APMV-6 (Table 1 and Table S7). In general, high-quality complete genomes were obtained by nanopore sequencing on a MinION device in veterinary labs in Ukraine. APMV-1/Mallard/Myt Kherson/1-4/4-09/17 had no coverage until position 885 along the genome and had adequate coverage (>25×) until position 14,530, where the coverage dropped to 0×, and a small region between 13,680 and 13,998 had an average 20× coverage; the remaining samples had adequate >25× coverage across the genome except for the ends in the noncoding regions (Figure 2, Figures S1 and S2; Table S7). Genome assemblies indicated similar sizes typical for APMV-1 and APMV-6 (Figures S1 and S2; Table S7). Recombination analysis using RDP5 did not identify evidence of recombination in the APMV-1 or APMV-6 genomes sequenced in this study.

**Figure 3:**
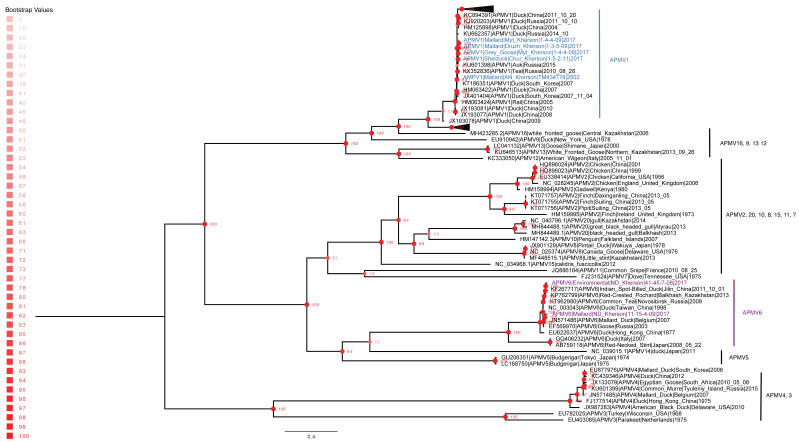
Global phylogenetic analysis comparing APMV-6 and APMV-1 with all avian avulavirus subtypes. Maximum likelihood tree showing evolutionary relationships among 322 full-length APMV sequences, including the seven sequenced in this study, and the phylogenetic position of Ukraine APMV-6 and APMV-1 sequences within their subtypes. APMV-1 strains sequenced in this study are highlighted in blue, and APMV-6 sequenced from this study are highlighted in purple. Eighty-nine full-lengthh segments are displayed, and the remaining 233 sequences are collapsed at the top of the tree. The collapsed sequences are all APMV-1 subtypes. Nodes indicate maximum likelihood bootstrap support under using bestTree and visualized in FigTree v.1.4.4 with a midpoint tree root.

**Figure 4:**
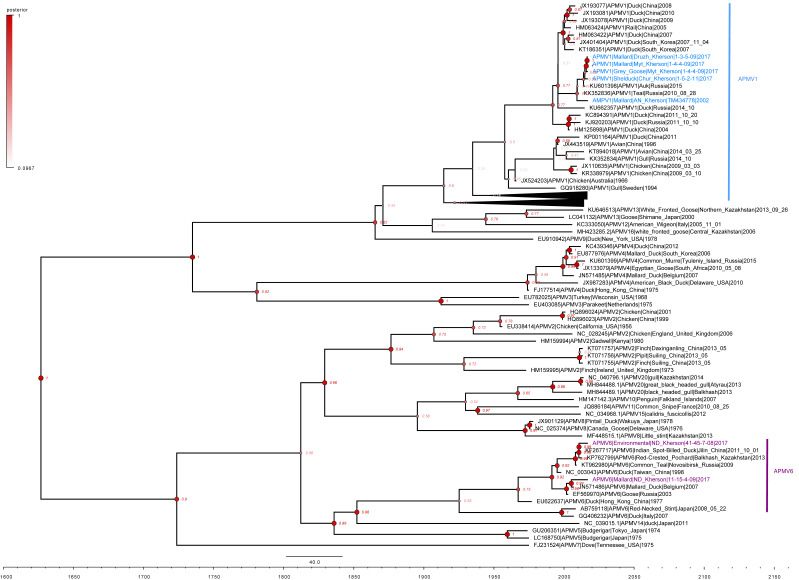
Phylogenetic analysis comparison of all APMV-1 and APMV-6 with APMV strains by a time-scale Bayesian maximum clade credibility tree of 315 APMV reference strains and seven isolated in Ukraine. APMV-1 strains sequenced in this study are highlighted in blue, and APMV-6 are highlighted in purple. Collapsed sequences are APMV-1 subtypes. Nodes indicate posterior probability support with a midpoint tree root.

The APMV-1 and APMV-6 genomes sequenced in this study were compared to 315 full-length avian avulaviruses with representatives from each subtype (except APMV-17, APMV-18, and APMV-19 due to a lack of publicly available full-length sequence data), through the construction of a maximum likelihood (ML) phylogenetic tree (Figure 3). Bayesian analysis of the same 322 sequenced genomes had a similar topology to the maximum likelihood analysis (Figure 4). Interpreting the phylogenies, the Ukrainian APMV-1 and APMV-6 sequences showed the highest similarity to the reference sequences used in the genome assembly. The APMV-1 viruses isolated from Ukraine clustered with duck and other wild Anseriformes viruses detected across Eurasia, with a subclade in Ukraine and Russia (ML bootstrap value, 98/100, 100 bootstraps; Bayesian posterior, 0.77) and two distinct sublineage branches (ML bootstrap value, 98/100, 100 bootstraps; Bayesian posterior, 0.71) that suggested at least two separate infection events (Figure 3 and Figure 4). Similarly, the APMV-6 viruses formed subclades that appeared to be the result of multiple distinct infection events (bootstrap value, 100; Bayesian posterior, 0.92) among ducks and wild Anseriformes originating in a broad group of APMV-6 sequences spanning Europe and Asia (Figure 4). These results suggested ecologically diverse APMV-1 and APMV-6 reservoirs in Eurasia, with multiple host species and broad geographic ranges.

## 4. Discussion

Avian influenza surveillance in the Azov-Black Sea region in Ukraine has opened the door for the detection of other avian pathogens such as APMV. A total of 4790 fecal samples from a variety of birds, predominately from wild waterfowl and shorebirds (the Anseriformes and Charadiformes orders), were screened for AIV and APMV. In Ukraine, the mean paramyxovirus isolation rate ranged from 0.26 to 1.94% in a variety of Anseriformes species (greylag goose, mallard duck, common shelduck, and ruddy shelduck; Table S2) which is low in comparison with the 2006–2011 sampling seasons [12]. Muzyka et al. (2014) detected a seasonality effect in the isolation rate, so further analyses will identify whether there was a seasonality effect during the 2017 season [12]. Eleven samples tested positive for APMV-1 based on a 121 nt region in the matrix protein. The PCR-positive samples were found in wild birds of the Anseriformes (*n* = 8), Chardiiformes (*n* = 2) and Pelecaniformes (*n* = 1) orders (Table S3). The mean infection rate (by PCR detection) of APMV-1 across species was relatively low, except in white pelicans, who had a 2.43% mean infection rate. Large outbreaks of virulent APMV have led to large die-offs in cormorants and gulls, so infection in white pelicans in marine or coastal habitats is not surprising [43,44]. The circulation of APMV in gulls and pelicans can facilitate transmission if they are near poultry or other domesticated fowl farms [44].

For APMV, RT-PCR-based diagnostics are the most cost-effective and efficient means of identifying the virus; however, the hemagglutinin inhibition test has traditionally been used to determine the subtype. Eleven isolates tested positive for avian avulavirus (APMV1 *n* =6; APMV-4 *n* = 4; APMV-6 *n* = 2; APMV-1/7 *n* = 7), which is consistent with previous reports in Ukraine [12,20].

We used full genome sequencing of selected APMV-1 and APMV-6 positive samples to resolve ambiguities in the HI assay and genetic subtypes of the detected APMV serotypes. As a mainstay of capacity-building projects, a tiling primer approach combined with nanopore technology has proven successful in low-resourced laboratories. For example, this approach has been used to generate highly accurate sequences of Ebola and Zika viruses in outbreak settings and SARS-CoV-2 and other pathogens in Ukraine [29,30,45]. This study was the first report of the genome of an APMV-6 isolate from wild birds in Ukraine and the first use of our tiling and nanopore technology for APMV-1 and APMV-6 sequencing in sentinel veterinary labs in Ukraine. 

Seven full-length genomes were pre-amplified using a tiling primer RT-PCR approach and sequenced on MinION. For this study, we first tested our methodology in a well-resourced laboratory at the University of Alaska using an NDV La Sota vaccine strain. All seven APMV genomes were isolated from the host order Anseriformes: five APMV-1 genomes were isolated from mallards (*n* = 2), shelducks (*n* = 2) and a graylag goose (*n* = 1), and an additional two APMV-6 genomes were sequenced from a mallard and from an unidentified, fecal (environmental) sample, respectively. For one of the five APMV-1 sequences (APMV-1/Mallard/Myt Kherson/1-4/4-09/17), the 3′ end of the genome was not assembled, most likely due to insufficient amplification of this region. Four APMV-1 genomes (aside from APMV-1/Mallard/Myt Kherson/1-4/4-09/17) ranged in length from 15,085–15,168 nt. APMV-6 genome length ranged in size from 16,234–16,235 nt and were consistent with other APMV-6 genomes reported.

Upon further analysis of APMV-1 genomes, virulent strains of APMV-1 depended on the proteolytic cleavage of a multi-basic amino acid motif in the fusion protein that facilitated furin-like proteases to stimulate proteolytic cleavage and activation from the host cell [2,46]. Samples sequenced in the study displayed a mono-basic site, suggesting that all strains were avirulent [2]. While the viruses sequenced in this study were avirulent, a mild respiratory infection could still impact a flock and cause economic stress [47]. A previous evaluation of APMV-6 genomes suggested infections in chickens are asymptomatic or mild in other species [14,48]. Xiao et al. (2010) analyzed a GAGGGGGAAG motif located upstream of each protein in the untranslated region except for a matrix protein that had a GAGGGGGAAC motif. This was consistent with the APMV-6 genomes sequenced in this study [14]. Motifs downstream of each protein were conserved among APMV-6 strains and were found to be consistent in this study [14].

Phylogenetic analysis revealed that all five Ukrainian APMV-1 isolates were closely related to APMV-1 sequences from other wild birds in Eurasia: a large auk (*Uria aalge*) in Tyuleny Island in the Sea of Okhotsk and a Eurasian teal (*Anas crecca*) from southwestern Siberia (Russian Federation). In addition, the Ukrainian APMV-1 isolates were also phylogenetically clustered with those sampled from mallards and a water rail (*Rallus aquaticus*) in South Korea and China. These bird species migrate annually between breeding grounds in Asia and the Siberian tundra and have natural habitats in southern Europe and Africa as part of the Black Sea–Mediterranean flyway route [49]. Therefore, it is likely that APMV viruses circulate annually in southern Ukraine, in particular in the Azov-Black Sea region, since many bird species use this region as a stopover on the flyway during migration periods. 

The reconstructed phylogeny was focused on evaluating the evolutionary history based on full-length APMV genomes; thus partial sequences previously obtained in Ukraine were not included in the analysis. The isolate APMV1/Mallard/Myt Kherson/1-4-4-09/2017 failed full-length assembly due to the low coverage depth at the beginning of the genome (3′-UTR region). Inadequate sequencing at the beginning and end of the genome in the noncoding region was consistent when sequencing other RNA viruses, such as Zika, using the tiling primer approach [29]. Newer, randomly primed, strand-switching amplification protocols have been recently developed that may complement tiling primer-based sequencing, with particular strength in the agnostic sequencing of divergent RNA viruses [50]. However, this protocol was not available at the time of sequencing in Ukraine, and remain to be robustly tested in low-resourced laboratories or field settings.

The reconstruction of evolutionary relationships for APMV-1 and APMV-6 were remarkably similar despite belonging to entirely different clades or groups. Evidence of circulation in wild water birds, primarily belonging to the Anseriformes originating from Eurasia, was a consistent pattern between APMV groups. The Bayesian analysis indicated a TMRCA (time to the most recent common ancestor) estimate that APMV-1 and APMV-6 emerged in the early 2000s, although historical inconsistency and the widespread ecogeography of APMV sampling make it difficult to clearly represent the temporal origin of the viruses.

The long-distance movement of wild birds connecting Ukraine to other regions, including Europe, Central Asia, and East Asia, plays an important role in the mixing of virus populations from Europe (west) and Asia (east), consistent with studies of AIV and APMV in wild bird reservoirs in the Azov-Black Sea wetland ecologies of southern Ukraine [12,19,20]. While both the maximum likelihood and Bayesian phylogenetic trees identified similar topologies, APMV sequences are likely under-sampled, given their prevalence. Robust sampling efforts are required to classify an emerging viral event in detail. Since APMV identification and isolation have largely been based on AIV surveillance efforts, there is a historical discrepancy between APMV sampling and sequencing, making it difficult to predict the exact location and timing of the emergence of the viruses sequenced in our study.

## 5. Conclusions

Building from longitudinal surveillance of avian disease pathogens in Ukraine, this study was the first to isolate and sequence seven full-length avian avulavirus (APMV) genomes from wild ducks and geese in the Azov-Black Sea region in Ukraine. Five novel APMV-1 and two APMV-6 genomes were sequenced and clustered with contemporary circulating strains in Russia, Europe, and East Asia, suggesting a broad but understudied reservoir and transmission pattern of these pathogens across the Eurasian continents.

## Figures and Tables

**Table 1:** APMV viruses isolated from Ukraine during the 2016–2017 field season. Both APMV-1 and APMV-6 were predominately isolated from the southern Oblasts of Ukraine. HI = hemagglutinin assay.

Sample Name	HI Test Results	Sample Description
APMV1|Mallard|Myt_Kherson|1-4-4-09|2017	APMV1	Genetic material (RNA) collected in September 2017 in Kherson Oblast from a clinically healthy wild mallard.
APMV1|Mallard|AN_Kherson|TM434778|2002	APMV1	Genetic material (RNA) collected in December 2002 in Kherson Oblast from a clinically healthy wild mallard.
APMV-6|Environmental|ND Kherson|41-45|7-08|2017	APMV6	Genetic material (RNA) collected in August 2017 in Kherson Oblast from the environment.
APMV-6|Mallard|ND Kherson|11-15|4-09|2017	APMV6	Genetic material (RNA) collected in September 2017 in Kherson Oblast from a clinically healthy wild mallard.
APMV-1|Mallard|Dr_Kherson|1-3|5-09|2017	APMV1	Genetic material (RNA) collected in September 2017 in Kherson Oblast from a clinically healthy wild mallard.
APMV-1|Grey_Goose|Myt_Kherson|1-4|4-09|2017	APMV1	Genetic material (RNA) collected in September 2017 in Kherson Oblast from a clinically healthy wild grey goose.
APMV-1|Shelduck|Chur_Kherson|1-5|2-11|2017	APMV1	Genetic material (RNA) collected in November 2017 in Kherson Oblast from a clinically healthy wild shelduck.

## Data Availability

Sequence data for AMPV-1 and APMV-6 genomes are available at NCBI GenBank under BioProject (PRJNA889548), accession numbers (OQ584346-OQ584352).

## Supplementary Materials

The following are available online at https://www.mdpi.com/article/10.3390/v15030699/s1, Supplementary Table S1: Avian sampling; Supplementary Table S2: Infection of APMV-1 in wild birds by RT-PCR; Supplementary Table S3: Isolation of APMV in ovo; Supplementary Table S4: Samples tested by HI assay from wild birds; Supplementary Table S5: APMV tiling primer sequences (spreadsheet .xlsx); Supplementary Table S6: APMV reference sequences used to build APMV reference database (.fasta); Supplementary Table S7: APMV-1 and APMV-6 samples sequenced; Supplementary Table S8–S14: Amino acid substitution tables for novel APMV-1 and APMV-6 in comparison to reference genomes; Supplementary Figure S1: Genome assembly of APMV-1 sequences; Supplementary Figure S2: Genome assembly of APMV-6 sequences.
