# Supplementary material for: Discovery of Avian Paramyxoviruses APMV-1 and APMV-6 in Shorebirds and Waterfowl in Southern Ukraine
Source: Viruses. 2023 Mar 8;15(3):699. doi: 10.3390/v15030699 (PMC10058161; doi:10.3390/v15030699)
Supplement: Supplementary file 1 [file viruses-15-00699-s001.zip › viruses-1796880-supplementary.pdf]

**Supplementary Tables and Figures**

**Supplementary Table S1.** Avian sampling. Complete list of fecal samples from wild bird species collected in both southern and northern Ukraine from December 2016 – December 2017. Sampling in the autumn (August-October) and winter (November-February) seasons were focused on collecting migrating birds, while spring season was focused on spring migration (March-May) and sampling prior to and after nesting period (June-July).

| **Bird Species** | | **Seasons** | | | |
|---|---|---|---|---|---|
| **Common Name** | **Scientific Name** | **Winter** | **Spring** | **Autumn** | |
| ***ANSERIFORMES*** | | | | | **Total** |
| White-fronted Goose | *Anser albifrons* | 417 | 257 | 359 | 1033 |
| Red-breasted Goose | *Rufibrenta ruficollis* | - | - | 23 | 23 |
| Garganey | *Anas querquedula* | 25 | 18 | | 43 |
| Mallard | *Anas platyrhynchos* | 253 | 415 | 432 | 1100 |
| Shelduck | *Tadorna tadorna* | 208 | 82 | 139 | 429 |
| Ruddy Shelduck | *Tadorna ferruginea* | 35 | 25 | 43 | 103 |
| Mute Swan | *Cygnus olor* | 11 | 23 | 15 | 49 |
| Whooper Swan | *Cygnus cygnus* | 125 | - | 145 | 270 |
| Greylag Goose | *Anser anser* | 32 | 340 | 14 | 386 |
| Wigeon | *Anas penelope* | 5 | - | 10 | 15 |
| Shoveler | *Anas clypeata* | 22 | 2 | - | 24 |
| Teal | *Anas crecca* | 10 | - | 51 | 61 |
| Wild duck | *Anas spp.* | 38 | - | - | 38 |
| Pintail | *Anas acuta* | 6 | - | - | 6 |
| ***CHARADRIIFORMES*** | | | | | |
| Yellow-legged Gull | *Larus cachinnans* | 23 | 48 | 62 | 133 |
| Black-headed Gull | *Larus ridibundus* | 8 | 236 | 18 | 262 |
| Slender-billed Gull | *Larus genei* | 20 | 16 | - | 36 |
| Ruff | *Phylomachus pugnax* | 101 | 1 | - | 102 |
| Mediterranean Gull | *Larus melanocephalus* | 20 | 169 | - | 189 |
| Common Gull | *Larus canus* | 10 | - | 8 | 18 |
| Great Black-headed Gull | *Larus ichtyaetus* | - | 5 | 35 | 40 |
| Dunlin | *Calidris alpina* | - | - | 10 | 10 |
| Snipe | *Gallinago gallinago* | - | - | 15 | 15 |
| Little Gull | *Larus minutus* | - | 5 | - | 5 |
| Little Tern | *Sterna albifrons* | - | 5 | - | 5 |
| White-winged Black Tern | *Chlidonias leucopterus* | - | 20 | - | 20 |
| Caspian Tern | *Hydroprogne caspia* | 7 | - | - | 7 |
| Avocet | *Recurvirostra avosetta* | 5 | - | - | 5 |
| Gulls spp. | | 4 | 10 | - | 14 |

| | | | | | |
|---|---|---|---|---|---|
| ***PODICIPEDIFORMES*** | | | | | |
| Great Crested Grebe | *Podiceps cristatus* | - | - | 1 | 1 |
| ***GRUIFORMES*** | | | | | |
| Demoiselle Crane | *Anthropoides virgo* | - | 12 | - | 12 |
| Crane | *Grus grus* | 16 | 17 | - | 33 |
| Coot | *Fulica atra* | 9 | - | - | 9 |
| ***CICONIIFORMES*** | | | | | |
| Great White Egret | *Egretta alba* | - | 15 | - | 15 |
| Little Egret | *Egretta garzetta* | - | 5 | - | 5 |
| Grey Heron | *Ardea cinerea* | - | 29 | - | 29 |
| Spoonbill | *Platalea leucorodia* | - | 7 | - | 7 |
| ***PELECANIFORMES*** | | | | | |
| Dalmatian Pelican | *Pelecanus crispus* | - | 13 | 2 | 15 |
| Cormorant | *Phalacrocorax carbo* | - | 64 | 67 | 131 |
| White Pelican | *Pelecanus onocrotalus* | 15 | 26 | - | 41 |
| Pygmy Cormorant | *Phalacrocorax pygmaeus* | 5 | - | - | 5 |
| **FALCONIFORMES** | | | | | |
| Marsh Harrier | *Circus aeruginosus* | - | 1 | - | 1 |
| ***OTHER*** | | | | | |
| Environmental (Fresh bird feces) | *Species was not identified* | - | 45 | - | 45 |
| **Total** | | **1430** | **1911** | **1449** | **4790** |

**Supplementary Table S2.** Overview of the infection rate of APMV-1 in wild birds by RT-PCR.

| Species | Infection Rate (%) |
|---|---|
| Whooper Swan | 0.37 |
| Mallard | 0.36 |
| Mediterranean Gull | 0.52 |
| Shelduck | 0.46 |
| White-fronted Goose | 0.09 |
| White pelican | 2.43 |
| Snipe | 0.07 |

**Supplementary Table S3.** The isolation rate of APMV (avulavirus) in wild birds by virus isolation *in ovo*.

| Species | Isolation Rate (%) APMV |
|---|---|
| Greylag Goose | 0.26 |
| Mallard | 0.54 |
| Shelduck | 0.46 |
| Ruddy shelduck | 1.94 |

**Supplementary Table S4.** Overview of samples tested by HI assay from wild birds in 2017. AIV, avian influenza virus with provisional HA serological subtype noted, APMV avian paramyxovirus (avulavirus) with provisional type noted; Unidentified, samples that were HI positive (hemagglutinating) but not identifiable by serological panel available.

| No. | Strain Name | Serological Identification |
|---|---|---|
| 1 | Shelduck/Baz Odesa/13-16-28-10/17 | AIV H2/H5/H7 |
| 2 | Mallard/Er Odesa/41-50-8-08/17 | AIV H3 |
| 3 | Mallard/Dr3 Kherson /1-5/6-08/17 | AIV H3 |
| 4 | Ruddy shelduck/Pop Kherson/1-5-23-01/17 | AIV H3 |
| 5 | Ruddy shelduck/Pop Kherson/6-10-23-01/17 | AIV H3 |
| 6 | Ruddy shelduck/Pop Kherson/11-15-23-01/17 | AIV H3 |
| 7 | Shelduck/Zh. Yar Odesa/1-5-27-10/17 | AIV H3/H7 |
| 8 | Environmental/ND Kherson/31-35-7-08/17 | AIV H4 |
| 9 | Mallard/Ol2 Kherson/33-36/5-09/17 | AIV H4 |
| 10 | Mallard/Dr3 Kherson /6-10-6-08/17 | AIV H4 |
| 11 | Mallard/Zh. Yar Odesa/1-5/10-08/17 | AIV H4 |
| 12 | Mallard/Syv Kherson/1-4/4-09/17 | AIV H5 |
| 13 | Mallard/Chon Kherson/13-16/3-09/17 | AIV H5 |
| 14 | Mallard/Hor Kherson/16-20/29-11/17 | AIV H7/H9 |
| 15 | Shelduck/Pop Kherson/21-25/23-01/17 | AIV H10 |
| 16 | Whooper Swan/Des Odesa/1-5/30-01/17 | AIV H11 |
| 17 | Whooper Swan/Des Odesa /6-10/30-01/17 | AIV H11 |
| 18 | Black-headed Gull / Dr3 Kherson /1-3/6-08/17 | AIV H11/H13 |
| 19 | Greylag Goose /Myt Kherson/1-4/4-09/17 | APMV-1 |
| 20 | Mallard/Myt Kherson/1-4/4-09/17 | APMV-1 |
| 21 | Shelduck/Ser Kherson/11-15/6-08/17 | APMV-1 |
| 22 | Mallard/Kat Odesa/1-5/1-12/17 | APMV-1 |
| 23 | Mallard/Kat Odesa/6-10/1-12/17 | APMV-1 |
| 24 | Shelduck/Chur Kherson/1-5/2-11/17 | APMV-1 |
| 25 | Ruddy shelduck/Hr Kherson/11-15/27-01/17 | APMV-1/APMV-7 |
| 26 | Mallard/Hor Kherson/56-60/29-11/17 | APMV-4 |
| 27 | Mallard/Druzh Kherson/1-3/5-09/17 | APMV-4 |
| 28 | Environmental/ND Kherson /41-45/7-08/17 | APMV-6 |
| 29 | Mallard/ND Kherson /11-15/4-09/17 | APMV-6 |
| 30 | Mallard/Chon Kherson/41-45/1-11/17 | AIV H1 |
| 31 | Ruddy shelduck/ ND Kherson /1-5/5-08/17 | Unidentified |
| 32 | Mallard/Prym T Odesa/35-39/26-09/17 | Unidentified |
| 33 | White fronted goose/Vas Kherson/2,4,5/29-11/17 | Unidentified |
| 34 | Environmental/ND Kherson /26-30/7-08/17 | Unidentified |
| 35 | Shelduck/Ser Kherson/16-20/6-08/17 | Unidentified |
| 36 | Mallard/Dr2 Kherson /16-20/6-08/17 | Unidentified |
| 37 | Mallard/Ras Odesa/36-40/11-08/17 | Unidentified |
| 38 | Mallard/Ras Odesa/16-20/11-08/17 | Unidentified |
| 39 | Mallard/PK Kherson/1-5/7-08/17 | Unidentified |
| 40 | Mallard/Vas Kherson/1-5/24-01/17 | Unidentified |

**Supplementary Table S7.** Overview of 2017 APMV-1 and APMV-6 positive samples sequenced using MinION. APMV was amplified using a RT PCR with four pools of tiling primers. Amplicons were sequenced with an LSK109 nanopore sequencing kit. Sequences were aligned to a reference database to identify a closest match for further analysis with Minimap2. Consensus sequences were generated using Medaka. Sequences were realigned to the reference and the percent identity was calculated with Geneious.

| Sample Identifier | Species | Sample Location | Total # of Reads Sequenced | Closest Reference | Percent Identity | Consensus Sequence Length |
|---|---|---|---|---|---|---|
| APMV1\|Mallard\|Myt_Kherson\|1-4-4-09\|2017 | Mallard | Myt Kherson | 92,095 | KX352836\|APMV1\|Teal\|Russia\|2010_08_28 | 98.4% | 14,089 |
| APMV1\|Mallard\|AN_Kherson\|TM434778\|2002 | Mallard | Kherson | 653,240 | KX352836\|APMV1\|Teal\|Russia\|2010_08_28 | 98.7% | 15,168 |
| APMV-6\|Environmental\|ND Kherson\|41-45\|7-08\|2017 | Env. | Kherson | 429,606 | JX522537.1\|APMV6\|Mallard\|China\|2011 | 98.6% | 16,234 |
| APMV-6\|Mallard\|ND Kherson\|11-15\|4-09\|2017 | Mallard | Kherson | 764,971 | JN571486\|APM6\|Duck\|Belgium\|2007 | 96.6% | 16,235 |
| APMV-1\|Mallard\|Dr_Kherson\|1-3\|5-09\|2017 | Mallard | Druzh Kherson | 121,493 | KU601398\|APMV1\|Auk\|Russia\|2015 | 98.7% | 15,085 |
| APMV-1\|Grey_Goose\|Myt\|1-4\|4-09\|2017 | Grey goose | Myt Kherson | 925,542 | KU601398\|APMV1\|Auk\|Russia\|2015 | 99.1% | 15,093 |
| APMV-1\|Shelduck\|Chur_Kherson\|1-5\|2-11\|2017 | Shelduck | Chur Kherson | 1,276,200 | KU601398\|APMV1\|Auk\|Russia\|2015 | 99.2% | 15,090 |

**Supplementary Table S8.** Amino acid substitution table for APMV1/Mallard/Myt Kherson/1-4/4-09/17 compared to reference KX352836|APMV1|Teal|Russia|2010_08_28.

| Nucleoprotein | Phosphoprotein | Matrix Protein | Fusion Protein | Hemagglutinin Protein | Large Polymerase Protein |
|---|---|---|---|---|---|
| Y230H | T210I | K226R | None | L211P | S3G |
| | S239N | G230R | | RR 293G | V187I |
| | I248V | | | E495V | V207E |
| | A341V | | | C596Y | I661V |
| | M342I | | | K603E | S894F |
| | | | | | S952N |
| | | | | | R1071K |
| | | | | | N1564H |
| | | | | | S1753N |
| | | | | | weird |
| | | | | | weird |
| | | | | | weird |
| | | | | | T2095I |
| | | | | | L2139P |
| | | | | | weird |
| | | | | | weird |
| | | | | | D2053N |
| | | | | | weird |

**Supplementary Table S9.** Amino acid substitution table for APMV-1/Mallard/AN Kherson/TM434778/2002 compared to reference KX352836|APMV1|Teal|Russia|2010_08_28.

| Nucleoprotein | Phosphoprotein | Matrix Protein | Fusion Protein | Hemagglutinin Protein | Large Polymerase Protein |
|---|---|---|---|---|---|
| None | A78V | None | T203A | R63K | S3G |
| | | | | C123S | S156C |
| | | | | D285E | V187I |
| | | | | R293G | O626P |
| | | | | K567R | S894F |
| | | | | I590V | E1031G |
| | | | | K603E | P1734S |
| | | | | | Y2202C |

**Supplementary Table S10.** Amino acid substitution table for APMV-6/Environmental/ND Kherson/41-45/7-08/17 compared to reference JX522537.1|APMV6|Mallard|China|2011.

| Nucleoprotein | Hemagglutinin Protein | Phosphoprotein | Matrix Protein | Fusion Protein | Large Polymerase Protein | SH Protein |
|---|---|---|---|---|---|---|
| A79T | K57R | S64P | V342I | N20S | T51A | T49A |
| D419N | P64S | S68P | | A76T | I121V | N89T |
| | S71G | N83D | | S77G | A405T | |
| | V171A | H168Y | | E148D | S637N | |
| | V212I | | | | P650L/M | |
| | R356G | | | | H725N | |
| | C438T | | | | P939L | |
| | I446L/M | | | | Q1097H | |
| | A481V | | | | N1269K | |
| | F560S | | | | Y1599C | |
| | T604A | | | | V2031A | |
| | | | | | Q2099H | |
| | | | | | L2229F | |

**Supplementary Table S11.** Amino acid substitution table for APMV-6/Mallard/ND Kherson/11-15/4-09/17 compared to reference JN571486|APM6|Duck|Belgium|2007.

| Nucleoprotein | Hemagglutinin Protein | Phosphoprotein | Matrix Protein | Fusion Protein | Large Polymerase Protein | SH Protein |
|---|---|---|---|---|---|---|
| G50S | V34I | T60I | I48V | L8P | R131Q | V20G |
| | I63V | S78I | | R26G | N176S | I38V |
| | E106K | G82E | | E98D | R207K | G39R |
| | N153S | S84L | | | E294G | N90D |
| | V205A | P94L | | | E322D | |
| | V226M | P108L/M | | | I456V | |
| | D293N | T117X | | | K502R | |
| | Q315H | N118H | | | L631F | |
| | L339F | T150A | | | T643K | |
| | K414R | A166T | | | P644S | |
| | G596M | T175A | | | T648A | |
| | Y603H | D196E | | | D726N | |
| | | E206D | | | P937L | |
| | | F246L | | | L1059Q | |
| | | A348T | | | S1400I | |
| | | I379T | | | N1814H | |
| | | T380M | | | P2080S | |
| | | N415S | | | | |

**Supplementary Table S12.** Amino acid substitution table for APMV-1/mallard/Dr Kherson/1-3/5-09/17 2017 compared to reference KU601398|APMV1|Auk|Russia|2015.

| Nucleoprotein | Hemagglutinin Protein | Phosphoprotein | Matrix Protein | Fusion Protein | Large Polymerase Protein |
|---|---|---|---|---|---|
| Y489H | L211P | V78A | S48L | I335T | K263R |
| | I270M | S239N | K256R | | I417V |
| | E495V | I248V | G230R | | O626P |
| | C596Y | A341V | | | I661V |
| | A598T | M342I | | | S952N |
| | | | | | V1365M |
| | | | | | N1564H |

**Supplementary Table S13.** Amino acid substitution table for APMV-1/Grey goose/Myt/1-4/4-09/17 compared to reference KU601398|APMV1|Auk|Russia|2015.

| Nucleoprotein | Hemagglutinin Protein | Phosphoprotein | Matrix Protein | Fusion Protein | Large Polymerase Protein |
|---|---|---|---|---|---|
| Y489H | I270M | V78A | K226R | I335T | K263R |
| | E495K | T210I | G230R | | I417V |
| | C596Y | S239N | | | O626P |
| | A598T | A341V | | | I661V |
| | | M342I | | | S952N |
| | | | | | V1365M |

**Supplementary Table S14.** Amino acid substitution table for APMV-1/shelduck/Chur/1-5/2-11/17 compared to reference KU601398|APMV1|Auk|Russia|2015.

| Nucleoprotein | Hemagglutinin Protein | Phosphoprotein | Matrix Protein | Fusion Protein | Large Polymerase Protein |
|---|---|---|---|---|---|
| N463S | I270M | none | T74I | V19A | T253M |
| | A598T | | | I335T | I417V |
| | | | | | K1071R |
| | | | | | V1365M |
| | | | | | M2056T |

| Sample ID | Nucleoprotein | Phosphoprotein | Matrix protein | Fusion protein | Hemagglutinin-Neuraminidase protein | Large polymerase protein |
|---|---|---|---|---|---|---|
| APMV1/Mallard/Myt Kherson/1-4-4-09/2017 | Not enough sequence data | 991 – 2178 | 2394 – 3488 | 3648 – 5309 | 5516 - 7366 | Not enough sequence data |
| APMV1/Mallard/AN Kherson/TM434778/2002 | 109 - 1578 | 1874 - 3061 | 3277 – 4371 | 4532 - 6193 | 6400 - 8250 | 8369 - 14983 |
| APMV1/Mallard/Dr Kherson /1-3-5-09/2017 | 100 - 1569 | 1873 - 3060 | 3276 - 4370 | 4531 - 6192 | 6399 - 8249 | 8268 - 14983 |
| APMV1/Grey Goose/Myt/1-4-4-09/2017 | 115 - 1584 | 1879 - 3066 | 3282 - 4376 | 4536 - 6197 | 6404 - 8254 | 8373 – 14987 |
| APMV1/Shelduck/Chur/1-5-2-11/2017 | 113 - 1582 | 1878 - 3065 | 3281 - 4375 | 4535 - 6196 | 6403 - 8253 | 8372 - 14985 |

**Supplementary Figure S1.** Genome assembly of APMV-1 sequences isolated in this study. Reads were aligned to APMV-1 specific reference database to identify a closest reference using Minimap2 and a consensus sequence was generated using Medaka. Consensus sequence was re-aligned to the reference to generate genome annotations using Geneious v.11.0.3.

| Sample ID | Nucleoprotein | Phosphoprotein | Matrix protein | Fusion protein | Small Hydrophobic Protein | Hemagglutinin – Neuraminidase Protein | Large polymerase protein |
|---|---|---|---|---|---|---|---|
| APMV6/Environmental/ND Kherson/41-45-7-08/2017 | 130 - 1408 | 1685 - 2977 | 3233 - 4333 | 4596 - 6263 | 6539 - 6965 | 7117 - 8958 | 9273 - 15998 |
| APMV6/Mallard/ND Kherson/11-15-4-09/2017 | 128 - 1525 | 1687 - 2978 | 3234 – 4334 | 4597 – 6264 | 6541 – 6969 | 7121 – 8962 | 9277 - 16002 |

**Supplementary Figure S2.** Genome assembly of APMV-6 sequences isolated from this study. Reads were aligned to APMV-6 specific reference database to identify a closest reference using Minimap2 and a consensus sequence was generated using Medaka. Consensus sequence was re-aligned to the reference to generate genome annotations using Geneious v11.0.3.
